# Investigating the pathways of medication adherence in renal transplant recipients based on the COM-B model: the parallel mediating effects of beliefs and emotion regulation efficacy 

**DOI:** 10.3389/fphar.2025.1702618

**Published:** 2025-12-01

**Authors:** Lili Zhou, Ke Cheng, Linbin Chen, San Han, Jingjing Wan

**Affiliations:** 1 Central South University Xiangya School of Nursing, Changsha, China; 2 Nursing Department, Outpatient and Emergency Operating Room, The Third Xiangya Hospital, Central South University, Changsha, Hunan, China

**Keywords:** kidney transplant recipients, medication adherence, medication literacy, social support, regulatory emotionalself-efficacy, beliefs about medication, mediation analysis

## Abstract

**Background:**

Suboptimal medication adherence remains a major cause of allograft failure after kidney transplantation. Previous studies have focused on isolated factors rather than integrated mechanisms. Based on the COM-B model, this study investigates the mediating roles of medication beliefs and regulatory emotional self-efficacy (RESE) between medication literacy, social support, and medication adherence.

**Methods:**

A cross-sectional survey included 351 kidney transplant recipients (KTRs) from a tertiary hospital in Changsha (April-July 2025). Participants completed a general information questionnaire, the Basel Assessment of Adherence to Immunosuppressive Medications Scale, the Chinese version of the RESE Scale, the Social Support Rating Scale, the Chinese Medication Literacy Scale, and the Beliefs about Medicines Questionnaire-Specific. Data were analyzed using SPSS and AMOS for descriptive, correlational, hierarchical regression, and mediation analyses (bootstrapping with 5000 samples).

**Results:**

The medication non-adherence rate in KTRs was 37.6%, primarily due to missed doses (33.3%). Medication literacy, social support, medication beliefs, and RESE were significantly correlated with adherence (*p < 0.01*). After controlling for demographic variables, these factors explained 47.2% of the variance in adherence. Path analysis showed that medication literacy (*β= -0.219*) and social support (*β= -0.180*) directly reduced non-adherence and also indirectly improved adherence through medication beliefs and RESE. Specifically, medication literacy had indirect effects via medication beliefs (*β= -0.034, 11.6%*) and RESE (*β= -0.039, 13.4%*); social support exerted indirect effects through medication beliefs (*β= -0.113, 35.0%*) and RESE (*β= -0.030, 9.3%*). All bootstrap 95% CIs excluded zero.

**Conclusion:**

Medication adherence among KTRs remains suboptimal. Within the COM-B framework, this study confirms that medication literacy and social support not only directly affect adherence but also exert indirect effects through the dual mediating pathways of medication beliefs and RESE. These findings suggest that clinical interventions should adopt a multidimensional approach, focusing not only on enhancing medication knowledge and support systems but also specifically addressing patients’ medication beliefs and emotional self-efficacy. A multi-path synergistic strategy is recommended to optimize intervention effectiveness.

## 1 Introduction

Kidney transplantation (KT) is the optimal treatment for end-stage renal disease. However, long-term outcomes remain suboptimal, with a 10-year graft survival rate of only 67.8%, significantly lower than the 96%–98% observed in the first year post-transplantation (Poggio et al., 2021; De Weerd and Betjes, 2018). Poor medication adherence is a key independent risk factor contributing to this disparity. Studies indicate that approximately 20%–70% of kidney transplant recipients (KTRs) exhibit non-adherence to immunosuppressive medications (IM), a prevalence higher than that observed in other solid organ transplant populations (Paterson et al., 2018; Gokoel et al., 2020; Zhou et al., 2025; Mathes et al., 2017). More critically, medication non-adherence increases the risk of graft failure by sevenfold and elevates mortality risk by threefold (Mathes et al., 2017). Hence, improving adherence is crucial for enhancing patient outcomes.

Medication adherence, defined as the extent to which a patient’s medication-taking behavior aligns with prescribed recommendations, is a critical component in quality control across disease screening, treatment, and long-term management (Kiliç et al., 2024; Wu et al., 2024; Huang et al., 2022). It directly influences both individual health outcomes and healthcare resource utilization. However, adherence is influenced by multiple factors, which the World Health Organization (WHO) categorizes into five dimensions: socioeconomic, therapy-related, condition-related, patient-related, and healthcare system-related factors (Burkhart and Sabaté, 2003). Among these, psychosocial and behavioral factors at the patient level are more amenable to intervention than other determinants, making them a primary focus of research. Specifically:

Medication literacy, derived from the broader concept of health literacy, refers specifically to an individual’s ability to understand and apply medication-related information to make appropriate treatment decisions (Pouliot et al., 2018). In patients with chronic conditions such as hypertension and coronary heart disease, poor medication literacy impedes correct medication use and often leads to reduced adherence due to suboptimal treatment outcomes (Ng et al., 2022; Zheng et al., 2019; Shen et al., 2020). Similarly, KTRs require lifelong administration of multiple medications, necessitating a high level of medication literacy. However, how medication literacy influences adherence toIM in this distinct population, as well as the underlying mechanisms involved, remains to be fully elucidated.

Medication beliefs refer to patients’ cognitive balance between the perceived necessity of treatment and concerns about potential side effects, serving as an independent predictor of adherence. Extensive research indicates that patients with stronger perceived necessity and fewer medication concerns generally exhibit better adherence (Ng et al., 2022; Wang et al., 2020; Zheng et al., 2019). Therefore, assessing and addressing medication beliefs in KTRs is essential to promoting long-term adherence behavior.

Social support encompasses the subjective support (emotional experience), objective support (material assistance), and support utilization (the extent to which individuals mobilize their social networks to seek and accept help) available to an individual. A robust support system encourages active engagement in treatment, which includes not only professional guidance from healthcare providers, such as medication counseling and side-effect management, but also practical supervision and reminders from family members (Wang et al., 2023; Steeb et al., 2019; Shahin et al., 2021).

Regulatory Emotional Self-Efficacy (RESE) refers to an individual’s confidence in their ability to manage negative emotions (such as frustration or anger) and express positive emotions when facing medication-related challenges or emotional fluctuations. In the context of transplantation, potential adverse drug effects, financial burdens, and uncertainties regarding long-term treatment outcomes can evoke anxiety and fear in patients, often leading to intentional non-adherence. Studies indicate that strong RESE helps patients better cope with illness-related stress and serves as a key psychological factor in addressing non-adherent behaviors (Hasking et al., 2018; Wilson et al., 2020; Liu et al., 2021).

Notably, although these factors have been demonstrated to correlate with adherence, their underlying pathways of influence remain unclear. In particular, the interplay of multiple factors and how they collectively affect medication-taking behavior hinders the development of systematic intervention strategies.

To elucidate these mechanisms, this study adopts the COM-B (Capability, Opportunity, Motivation–Behavior) model of behavior change as a theoretical framework (Michie et al., 2011). This model posits that behavior is directly influenced by capability, opportunity, and motivation, while capability and opportunity can also indirectly affect behavior through motivation. In the present study, medication literacy represents capability, social support represents opportunity, and medication beliefs along with RESE collectively constitute the motivation component.

Medication beliefs and RESE may serve as key mediators linking medication literacy and social support to medication adherence, a proposition supported by multiple empirical studies.

Specifically, regarding the mediating pathway of medication beliefs, the mechanism aligns closely with the theoretical expectations of the necessity–concerns framework (Horne and Weinman 1999). This model posits that influencing factors such as medication literacy and social support can affect adherence both directly and indirectly through medication beliefs. Medication beliefs play a significant mediating role in the relationship between medication literacy and adherence. For instance, Zhang et al. (2024) found that medication beliefs mediated 19.46% of this relationship among elderly stroke patients. Similarly, Neiva Pantuzza et al. (2022) demonstrated that improving medication knowledge—a core component of medication literacy—helps reduce patients’ concerns about medicines, thereby decreasing non-adherence.

A comparable mediating mechanism exists in the effect of social support on adherence. Chen Y. et al. (2023) reported that medication beliefs mediated 6.51% of this relationship in patients with schizophrenia, consistent with other studies indicating that support from family and healthcare providers strengthens patients’ belief in the benefits of drug therapy, thus improving adherence (Deng et al., 2023; Karazeybek, 2025; Gong et al., 2024).

In terms of the RESE pathway, self-efficacy-related variables—with RESE as a core dimension—also show significant mediation effects. Shen et al. (2020) observed that self-efficacy mediated 28.7% of the relationship between medication literacy and adherence in hypertensive patients. Wang et al. (2024) further reported that inner strength, reflecting emotional regulation ability, mediated 13.22% of the effect of medication literacy on adherence in KTRs.

Moreover, as an external resource, social support may indirectly promote adherence by enhancing psychological regulation. Given that RESE operates at the individual psychological level, this pathway is theoretically plausible. Studies by Yang et al. (2022) and Wu et al. (2018) further support this, showing that emotional support from family and peers enhances patients’ ability to regulate emotions such as anxiety and depression, thereby helping maintain higher medication adherence in stem cell and liver transplant recipients.

**FIGURE 1:**
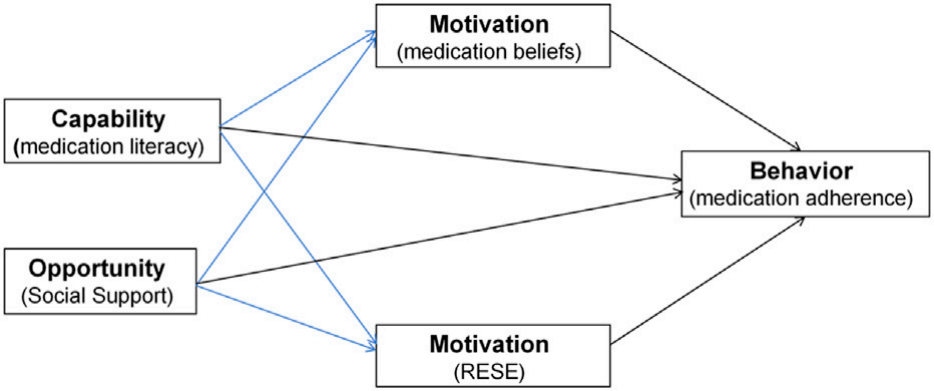
Conceptual framework. Blue lines represent indirect effects, and black lines represent direct effects.

In summary, although medication beliefs and RESE have been supported as mediators in some studies, evidence in KTRs remains limited. More importantly, whether medication beliefs and RESE act as parallel mediators in KTRs has not been systematically examined within a unified theoretical framework. Based on this, the present study proposes the following hypotheses: 1) Medication literacy, social support, medication beliefs, and RESE are significantly associated with medication adherence in KTRs. 2) Medication beliefs and RESE serve as parallel mediators between medication literacy, social support, and medication adherence. To enhance theoretical clarity, the conceptual framework of this study is presented in Figure 1.

## 2 Materials and methods

### 2.1 Design and setting

This study employed a cross-sectional survey design and was conducted at the Organ Transplant Center of the Third Xiangya Hospital, Central South University, in Changsha, Hunan Province. Ethical approval was obtained from the Ethics Committee of the Xiangya School of Nursing, Central South University, in January 2025 (Approval No.: E202530).

### 2.2 Participants

A total of 351 KTRs were recruited from the transplant center between April and July 2025. The inclusion criteria were as follows: (1) age ≥18 years; (2) functioning renal transplant (not on dialysis); (3) provision of informed consent and voluntary participation; (4) no psychiatric disorders or other severe physical illnesses. Exclusion criteria included (1) recipients of two or more organ transplants; (2) presence of cognitive or communication impairments; and (3) withdrawal from the study or incomplete questionnaire responses.

### 2.3 Methodological details and procedure

After being informed of the study’s purpose, procedures, and anonymity policy, eligible KTRs provided written informed consent to participate. All investigators received standardized training and passed a qualification assessment. Data were collected via self-administered paper-based questionnaires distributed on-site. The research staff primarily handled questionnaire distribution and collection and performed immediate checks for completeness upon retrieval. All questionnaires were completed independently by participants. A total of 360 questionnaires were distributed; 9 were excluded due to incomplete or patterned responses, resulting in 351 valid questionnaires and an effective response rate of 97.5%.

### 2.4 Survey questionnaires

#### 2.4.1 Demographic and clinical characteristics

A self-designed general information form was used to collect (1) sociodemographic data, including gender, age, marital status, education level, and current employment status; and (2) clinical characteristics, such as graft source, number of transplants, time since transplantation, duration of pre-transplant dialysis, number of medications, and post-transplant complications.

Number of medications refers to the total count of distinct prescription drugs taken daily by KTRs at the time of assessment. This includes both the core immunosuppressive regimen—such as tacrolimus, mycophenolate, and prednisone—as well as adjunct medications used to manage comorbidities, such as antihypertensives, hypoglycemic agents, and lipid-lowering drugs.

Post-transplantation complications in this study specifically refer to a range of clinical issues arising from immunosuppressive therapy, termed “medication-related post-KT complications.” (Kidney Disease: Improving Global Outcomes Transplant Work Group, 2009; Ketteler et al., 2018) Based on the literature review and expert clinical consultation, these complications were operationalized into eight categories for recording and statistical analysis (see Supplementary Table S3).

Medication adherence in this research specifically denotes adherence to IM medications.

#### 2.4.2 Chinese version of the medication literacy scale (C-MLS)

This scale, originally developed by Sauceda et al. (2012), assesses patients’ medication information processing and practical skills through simulated clinical scenarios. The Chinese version, adapted by Zheng et al. (2015), consists of 14 dichotomous items, with total scores ranging from 0 to 14. Scores below 4 indicate inadequate ability, scores between 4 and 10 reflect moderate ability, and scores above 10 represent good ability. The original adaptation study reported a test-retest reliability of 0.885 and a split-half reliability of 0.840.

This scale was selected because its scenario-based approach effectively captures KTRs’ real-world medication management abilities—rather than mere theoretical knowledge—making it well-aligned with the “Capability” construct of the COM-B model. In our sample, the scale demonstrated good internal consistency (Cronbach’s α = 0.780), and the data were suitable for factor analysis (KMO = 0.781, Bartlett’s χ^2^ = 1092.037, *p* < 0.001).

#### 2.4.3 Social support rating scale (SSRS)

This scale, developed by Xiao (1994) and colleagues within the Chinese sociocultural context, consists of 10 items grouped into three dimensions: subjective support, objective support, and utilization of support. Higher total scores indicate greater levels of social support, with scores ≤22 classified as low support, 23–44 as moderate support, and 45–66 as high support. This scale was selected for its three-dimensional structure, which comprehensively captures social support in the Chinese cultural setting. In the present study, the scale demonstrated good internal consistency, with an overall Cronbach’s α of 0.81 and subscale α values ranging from 0.71 to 0.81. Validity assessment confirmed the data’s suitability for factor analysis (KMO = 0.737, Bartlett’s χ^2^ = 1256.303, *p* < 0.001).

#### 2.4.4 Beliefs about medicines questionnaire-specific (BMQ-specific)

The scale, originally developed by Horne and Weinman (1999), is a well-established instrument for assessing patients’ medication beliefs. The Chinese version consists of 10 items rated on a 5-point Likert scale and comprises two dimensions: Necessity Beliefs and Concerns Beliefs. The total score is computed by subtracting Concerns scores from Necessity scores, with a positive value indicating stronger necessity beliefs. The original validation study reported good reliability and validity (Cronbach’s α = 0.77, S-CVI = 0.90) (Si et al., 2013). Its two-dimensional structure is directly based on the Necessity–Concerns Framework, making it highly suitable for examining the mediating role of medication beliefs in this study.

In the present study, Cronbach’s α for the total scale and its subscales ranged from 0.78 to 0.88. The data were suitable for factor analysis (KMO = 0.882, Bartlett’s χ^2^ = 1719.098, *p* < 0.001).

#### 2.4.5 Chinese version of the regulatory emotional self-efficacy scale (C-RESE)

The scale was originally developed by Caprara et al. (2008) and later adapted into Chinese through localization (Wen et al., 2009). It consists of 12 items across three dimensions: expressing positive emotions, regulating despondency or distress, and managing anger or irritability. Responses are rated on a 5-point Likert scale, with higher total scores indicating greater self-efficacy in emotion regulation. In the original Chinese version, the overall Cronbach’s α was 0.85, and subscale α values ranged from 0.77 to 0.85. This scale was selected because it effectively assesses KTRs’ ability to regulate negative emotions and maintain positivity under treatment-related stress, which aligns closely with the core focus of this study.

In the present study, the overall and dimensional Cronbach’s α values ranged from 0.70 to 0.85. The data were suitable for factor analysis, as indicated by a KMO value of 0.841 and Bartlett’s χ^2^ of 1069.990 (*p* < 0.001).

#### 2.4.6 Basel assessment of adherence with immunosuppressive medications scale (BAASIS)

This scale, developed specifically for transplant patients by Dobbels et al. (2010), assesses immunosuppressive medication adherence through five items. Items 1 to 4 evaluate missed doses, consecutive missed doses, timing adherence, and dose accuracy, each scored from 1 to 6. Item 5 records unauthorized discontinuation (yes/no). Adherence is defined as a total score of 4 on the first four items and a “no” response on Item 5; otherwise, non-adherence is indicated. The Chinese version demonstrates good reliability, with a Cronbach’s α of 0.697 and test-retest reliability of 0.964 (Shang et al., 2017). It was used as an outcome measure in this study due to its ability to identify various types of non-adherence and its international recognition.

In the present study, the scale exhibited acceptable internal consistency (Cronbach’s α = 0.70), and the data were suitable for factor analysis (KMO = 0.729, Bartlett’s χ^2^ = 343.863, *p* < 0.001).

### 2.5 Data analysis

Statistical analysis was performed using SPSS 27.0. Descriptive statistics were presented as means, standard deviations, frequencies, and percentages. Prior to inferential analyses, necessary statistical assumptions were verified. Normality of continuous variables was assessed using skewness and kurtosis coefficients, with absolute values below 3 and 8, respectively. Multicollinearity was examined using the variance inflation factor (VIF), and a VIF below 10 indicated acceptable collinearity (Kline, 2010).

Pearson correlation analysis was conducted to examine relationships among medication literacy, social support, medication beliefs, RESE, and medication adherence. Based on univariate analysis (*p* < 0.05), seven variables were selected as controls in a hierarchical regression analysis. Medication literacy, social support, medication beliefs, and RESE were subsequently entered to identify factors influencing IM adherence after KT. Common method bias was assessed using Harman’s single-factor test, with a result exceeding the recommended threshold indicating significant deviation.

Analysis of the indirect effects of medication beliefs and RESE on the relationship between medication literacy, social support, and medication adherence was performed using AMOS 28.0. The structural equation modeling (SEM) was conducted based on a covariance matrix, with parameters estimated via the maximum likelihood method. This approach was appropriate given the assumptions of normality and linearity, along with an adequate sample size for SEM. Mediation effects of medication beliefs and RESE were tested within the model. The bias-corrected percentile bootstrap method was applied to estimate 95% confidence intervals (95% CI) for the total, direct, and indirect effects. An effect was considered statistically significant if its 95% CI did not include zero, with a two-sided significance level set at *p* < 0.05 (Preacher and Hayes, 2004). The SEM was constructed with medication literacy and social support as independent variables, medication beliefs and RESE as mediators, and medication adherence as the dependent variable.

## 3 Results

### 3.1 Participant characteristics

Among the 351 participants, more than half were male (57.0%), with a mean age of 47.39 years (SD = 11.24). Approximately 31.9% had an education level of junior high school or below, 85.8% were married, and 49.9% were unemployed (Table 1).

**TABLE 1:** Characteristics of participants in this study (n = 351).

Variabes	N (%)
Gender	
Male	200 (57.0)
Female	151 (43.0)
Age	
18–44	147 (41.9)
45–59	143 (40.7)
≥60	61 (17.4)
Education level	
Junior school or below	112 (31.9)
High school	103 (29.3)
Junior college	66 (18.8)
Bachelor degree or above	70 (19.9)
Marital status	
Married	301 (85.8)
Unmarried/Divorced/Widowed	50 (14.2)
Employment status	
Employed	109 (31.1)
Retired	67 (19.1)
Unemployed	175 (49.9)
Kidney transplant source	
Living donor	154 (43.9)
Deceased donor	197 (56.1)
Number of transplants	
First transplant	332 (94.6)
Re-transplant	19 (5.4)
Post-transplant duration (years)	
<1	83 (23.6)
1–5	114 (32.5)
5–10	85 (24.2)
>10	69 (19.7)
Pre-transplant dialysis duration (years)	
<1	179 (51.0)
≥1	172 (49.0)
Number of medications	
≤3	19 (5.4)
>3	332 (94.6)
Daily medication frequency (doses)	
≤2	164 (46.7)
3–4	148 (42.2)
5–6	35 (10.0)
≥7	4 (1.1)
Postoperative complications	
Yes	250 (71.2)
No	101 (28.8)

### 3.2 Disease-related characteristics

A total of 56.1% of participants received kidneys from deceased donors, and 94.6% underwent their first KT. Dialysis duration was less than 1 year in 51.0% of the cohort, while 94.6% were taking more than three types of medications. Drug-related complications were reported by 71.2% of respondents. The mean post-transplantation time was 5.86 ± 5.68 years, with 32.5% of recipients having a transplant duration between 1 and 5 years (Table 1).

### 3.3 Scores for medication adherence, medication literacy, social support, medication beliefs, and RESE

The mean total score for medication adherence was 6.06 ± 1.89. A total of 132 participants (37.6%) exhibited non-adherent behavior. Among these, 117 individuals (33.3%) did not take their medication on time, instead taking it more than 2 h early or late. Seventy-three participants (20.8%) reported missing a single dose recently, 38 (10.8%) had missed two or more consecutive doses, 34 (9.7%) deviated from the prescribed dosage, and 6 (1.7%) discontinued their medication without medical authorization.

The mean total medication literacy score was 10.86 ± 2.30, indicating generally good medication literacy among participants. The mean total social support score was 40.81 ± 6.89, reflecting a moderate level of social support. The subjective support dimension received the highest average score (5.74 ± 1.27), while support utilization had the lowest (2.07 ± 0.50). The mean total score for medication beliefs was 6.35 ± 3.36. The average item score for necessity beliefs (3.94 ± 0.30) was higher than that for concerns beliefs (2.67 ± 0.61). The highest-scoring concern items were “I am worried about the long-term effects of my medicines” and “I am worried about becoming too dependent on my medicines.” The mean total RESE score was 46.03 ± 4.16. The POS subscale scored the highest (4.19 ± 0.44), while the ANG subscale scored the lowest (3.38 ± 0.55) (Table 2).

**TABLE 2:** Score of C-MLS, SSRS, BMQ-Specific, C-RESE and BAASIS for participants (n = 351).

Self-report instrument	Number of items	Range	Total score *(M±SD)*	Average item core*(M±SD)*	Ranking
C-MLS	14	7–14	10.86 ± 2.30	NA	-
SSRS	10	22–57	40.81 ± 6.89	4.08 ± 0.69	-
Subjective support	4	11–32	22.99 ± 5.07	5.74 ± 1.27	1
Objective support	3	4–28	11.60 ± 2.59	3.87 ± 0.86	2
Utilization of support	3	3–12	6.22 ± 1.50	2.07 ± 0.50	3
BMQ-specific	10	−2∼18	6.35 ± 3.36	3.31 ± 0.35	-
BMQ-necessity	5	14–25	19.72 ± 1.50	3.94 ± 0.30	1
BMQ-concerns	5	6–20	13.37 ± 3.06	2.67 ± 0.61	2
C-RESE	12	32–59	46.03 ± 4.16	3.84 ± 0.35	-
POS	4	9–20	16.75 ± 1.77	4.19 ± 0.44	1
DES	5	12–25	19.14 ± 1.85	3.83 ± 0.37	2
ANG	3	5–14	10.15 ± 1.66	3.38 ± 0.55	3
BAASIS	5	5–14	6.06 ± 1.89	NA	-

Footnote: *M* = mean, *SD*, standard, C-MLS, chinese version of the medication literacy scale; SSRS, social support rate scale, BMQ-Specific = Beliefs about Medicines Questionnaire-specific, BMQ-necessity = Evaluate patient attitudes toward the necessity of taking medication, BMQ-concerns = Evaluating patients’ concerns about taking medications, C-RESE, Chinese Version of the Regulation Self-efficacy Scale; POS, positive emotion; DES, Despondency/Distress, ANG, Anger/Irritation, BAASIS, Basel Assessment of Adherence with Immunosuppressive Medication Scale. NA = Not Applicable (Unidimensional dichotomous scale, scored 0 or 1).

### 3.4 Correlations among medication literacy, social support, medication beliefs, RESE, and medication adherence

Correlation analysis revealed that medication literacy, social support, medication beliefs, and RESE scores were all inversely correlated with medication adherence scores (*P < 0.01*). These correlations were in the expected direction: higher scores in medication literacy, social support, positive medication beliefs, and RESE were associated with lower medication adherence scores, indicating better adherence (Table 3). Additionally, medication literacy, social support, medication beliefs, and RESE were all positively correlated with each other (*P < 0.01*).

**TABLE 3:** Correlations among C-MLS, SSRS, BMQ-Specific, C-RESE and BAASIS for participants (n = 351, *r*).

Variables	BMQ-Specific	C-RESE	SSRS	C-MLS	BAASIS
BMQ-specific	1				
C-RESE	0.249**	1			
SSRS	0.410**	0.373**	1		
C-MLS	0.265**	0.414**	0.432**	1	
BAASIS	−0.472**	−0.359**	−0.447**	−0.429**	1

Footnote: **p < 0.01, C-MLS, Chinese Version of the Medication Literacy Scale; SSRS, social support rate scale, BMQ-Specific = Beliefs about Medicines Questionnaire-specific; C-RESE, Chinese Version of the Regulation Self-efficacy Scale; BAASIS, Basel Assessment of Adherence with Immunosuppressive Medication Scale.

### 3.5 Hierarchical multiple regression results

Variables including gender, KT source, number of transplantations, post-transplant duration, pre-transplant dialysis duration, number of medications, and postoperative complications were significantly associated with medication adherence levels (*P* < 0.05) and were therefore included as control variables in subsequent hierarchical multiple regression models (see Supplementary Table S4).

As shown in Tables 4, 5, in Model 1, which included only demographic and clinical characteristics, several variables were identified as significant predictors of medication adherence. Specifically, lower non-adherence was associated with having a living donor (β = −0.259, 95% CI [-1.397, −0.567]), a pre-transplant dialysis duration of less than 1 year (β = −0.250, 95% CI [-1.304, −0.581]), and taking more than three medications (β = 0.130, 95% CI [0.331, 1.837]). It is noteworthy that compared to patients with more than 10 years since transplantation, those with shorter post-transplant durations (<1 year, 1–5 years, 5–10 years) demonstrated significantly better medication adherence (β = −0.499, −0.299, −0.121, respectively; all 95% CIs excluded zero). This model explained 27.5% of the variance.

**TABLE 4:** Independent variables assignment of hierarchical multiple linear regression analysis.

Independent variables	Assignment
Gender	Male = 0; Female = 1
Kidney transplant source	Living donor = 0; deceased donor = 1
Number of transplants	First transplant = 0; Re-transplant = 1
Pre-transplant dialysis duration (years)	<1 = 0; ≥1 = 1
Number of medications	≤3 = 0; >3 = 1
Postoperative complications	Yes = 0; No = 1
Post-transplant duration (years)	<1 (*Z*_*1*_ *= 1 Z*_*2*_ *= 0 Z*_*3*_ *= 0 Z*_*4*_ *= 0*)
	1–5 (*Z*_*1*_ *= 0 Z*_*2*_ *= 1 Z*_*3*_ *= 0 Z*_*4*_ *= 0*)
	5–10 (*Z*_*1*_ *= 0 Z*_*2*_ *= 0 Z*_*3*_ *= 1 Z*_*4*_ *= 0*)
	>10 (*Z*_*1*_ *= 0 Z*_*2*_ *= 0 Z*_*3*_ *= 0 Z*_*4*_ *= 0*)
C-MLS	Continuous value
SSRS	Continuous value
BMQ-specific	Continuous value
C-RESE	Continuous value

Footnote: C-MLS, chinese version of the medication literacy scale; SSRS, social support rate scale, BMQ-Specific = Beliefs about Medicines Questionnaire-specific, C-RESE, Chinese Version of the Regulation Self-efficacy Scale.

**TABLE 5:** Hierarchical multiple linear regression analysis results.

Variables		Model 1(*β*)	Model 2(*β*)	Model 3(*β*)	Model 4(*β*)	Model 5(*β*)
		(95%CI)	(95%CI)	(95%CI)	(95%CI)	(95%CI)
Block1						
Gender		−0.081 (−0.653, 0.034)	−0.063 (−0.553, 0.072)	−0.047 (−0.482, 0.126)	−0.040 (−0.453, 0.146)	−0.037 (−0.437, 0.153)
Kidney transplant source		−0.259*** (−1.397, −0.567)	−0.180** (−1.066, −0.298)	−0.146** (−0.931, −0.18)	−0.138** (−0.895, −0.155)	−0.107* (−0.776, −0.035)
Number of transplants		0.001 (−0.744, 0.769)	0.028 (−0.460, 0.919)	0.031 (−0.413, 0.924)	0.035 (−0.368, 0.948)	0.035 (−0.358, 0.937)
Pre-transplant dialysis duration (years)		−0.250*** (−1.304, −0.581)	−0.252*** (−1.279, −0.622)	−0.229*** (−1.183, −0.542)	−0.208*** (−1.104, −0.467)	−0.180*** (−0.998, −0.359)
Number of medications		0.130 ** (0.331, 1.837)	0.135** (0.438, 1.808)	0.135** (0.456, 1.783)	0.136** (0.481, 1.786)	0.129** (0.430, 1.717)
Postoperative complications		0.084 (−0.112, 0.813)	0.092 (−0.039, 0.802)	0.122* (0.095, 0.916)	0.137** (0.165, 0.976)	0.161** (0.265, 1.072)
Post-transplant duration (years)	<1	−0.449*** (−2.527, −1.454)	−0.382*** (−2.188, −1.202)	−0.398*** (−2.241, −1.284)	−0.377*** (−2.144, −1.198)	−0.367*** (−2.094, −1.160)
	1–5	−0.299*** (−1.692, −0.709)	−0.243* (−1.429, −0.528)	−0.275*** (−1.545, −0.666)	−0.256*** (−1.463, −0.595)	−0.284*** (−1.575, −0.71)
	5–10	−0.121* (−1.051, −0.013)	−0.110*** (−0.957, −0.013)	−0.141** (−1.080, −0.158)	−0.131* (−1.030, −0.122)	−0.144** (−1.079, −0.183)
Block2						
BMQ-specific			−0.371*** (−0.256, −0.16)	−0.333*** (−0.234, −0.139)	−0.285*** (−0.209, −0.111)	−0.278*** (−0.204, −0.107)
Block3						
C-RESE				−0.212*** (−0.136, −0.057)	−0.174*** (−0.119, −0.039)	−0.143** (−0.105, −0.025)
Block4						
SSRS					−0.165** (−0.070, −0.020)	−0.127** (−0.060, −0.009)
Block5						
C-MLS						−0.171** (−0.221, −0.059)
*R* ^2^		0.294	0.417	0.455	0.474	0.491
ΔR^2^		0.275	0.400	0.437	0.455	0.472
F(*p*)		15.763***	24.367***	25.716***	25.380***	25.045***

Footnote: *p < 0.05, **p < 0.01, ***p < 0.001, *β* = Standardized coefficient, C-MLS, chinese version of the medication literacy scale; SSRS, social support rate scale, BMQ-Specific = Beliefs about Medicines Questionnaire-specific, C-RESE, Chinese Version of the Regulation Self-efficacy Scale.

After controlling for general demographic variables, Model 2 showed that medication beliefs significantly and negatively predicted non-adherence (β = −0.371, 95% CI [-0.256, −0.160]). In subsequent models, RESE (Model 3: β = −0.212, 95% CI [-0.136, −0.057]), social support (Model 4: β = −0.165, 95% CI [-0.070, −0.020]), and medication literacy (Model 5: β = −0.171, 95% CI [-0.221, −0.059]) each independently and negatively predicted non-adherence. These results indicate that higher levels of medication beliefs, RESE, social support, or medication literacy are associated with better medication adherence.

Furthermore, with the stepwise inclusion of these variables, the overall explanatory power of the models progressively increased. Beyond the general demographic controls, Models 2 through 5 contributed additional variances of 12.5%, 3.7%, 1.8%, and 1.7%, respectively. The final model accounted for a total of 47.2% of the variance in adherence. The overall fit of all five models was statistically significant (*P* < 0.001).

#### 3.5.1 Normality test

Normality of the included variables—medication literacy, medication beliefs, RESE, social support, and medication adherence—was assessed using SPSS version 27.0. The absolute values of skewness and kurtosis coefficients were all below 3 and 8, respectively, indicating that the data approximated a normal distribution. Detailed results are provided in Supplementary Table S1.

#### 3.5.2 Multicollinearity test

All five fitted models showed acceptable multicollinearity metrics, with tolerance values greater than 0.1 and VIFs below 5. These results confirm the absence of multicollinearity among the independent variables. See Supplementary Table S2 for details.

#### 3.5.3 Common method bias test

A total of 14 factors with eigenvalues greater than 1 were identified. The variance explained by the first principal component was 16.49%, which is well below the critical threshold of 40%. This indicates that no substantial common method bias was present in the data.

### 3.6 Path analysis results

**FIGURE 2:**
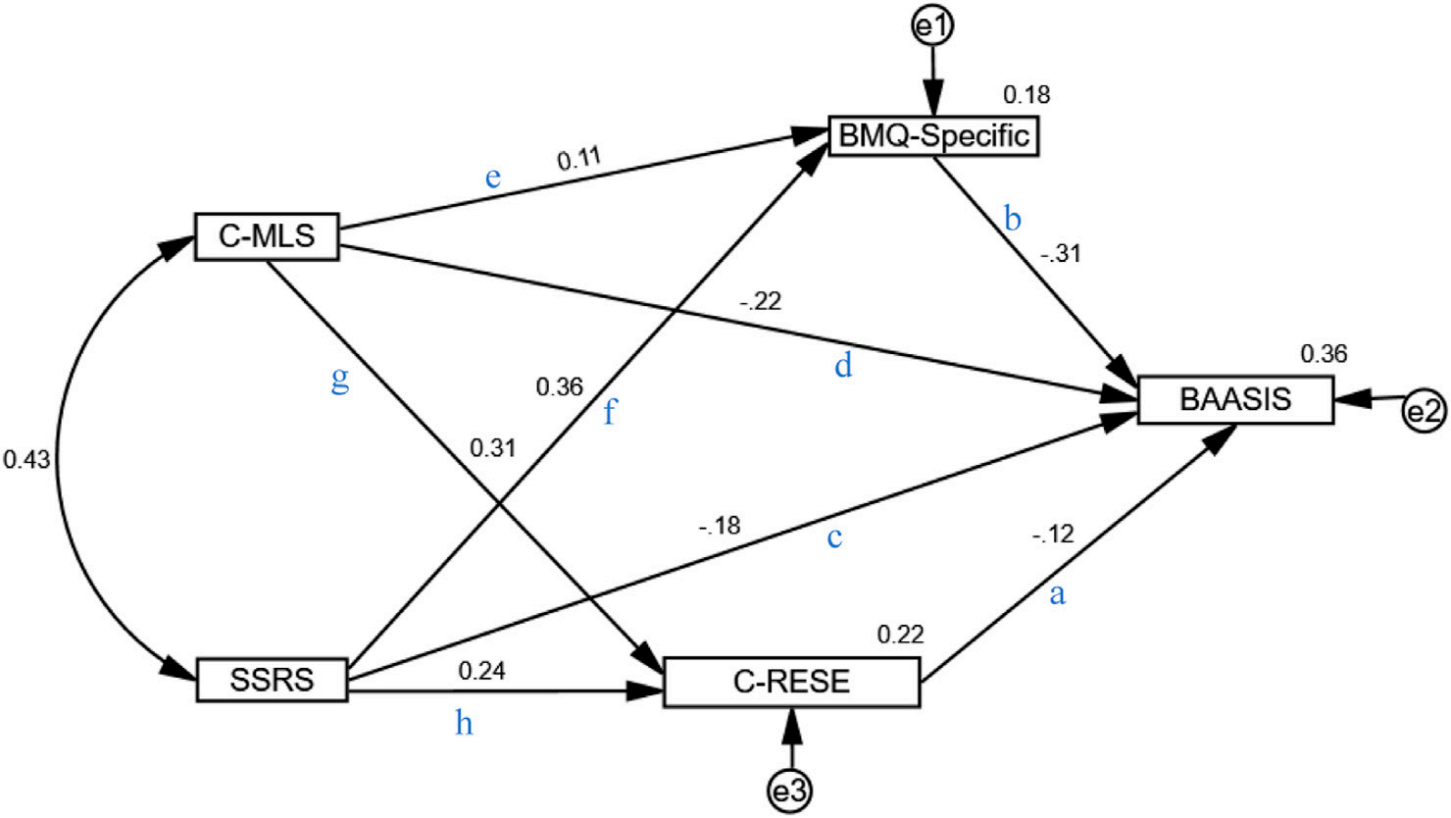
The mediation adherence model.

As shown in Figure 2, the initial model demonstrated a good fit to the data: χ^2^/df = 2.571, GFI = 0.997, AGFI = 0.956, RMSEA = 0.067, NFI = 0.993, RFI = 0.934, IFI = 0.996, TLI = 0.958, CFI = 0.996. All paths were statistically significant (*P* < 0.05). Medication beliefs exhibited the strongest direct effect on medication adherence (β = −0.310, *p* < 0.001), followed by medication literacy (β = −0.219, *p* < 0.001), social support (β = −0.180, *p* < 0.001), and RESE (β = −0.125, p = 0.001), as detailed in Table 6.

**TABLE 6:** Path coefficients of the structural equation model (n = 351).

Path label	Paths	*B*	*β*	SE	CR	*P*	Significance
a	C-RESE→BAASIS	−0.056	−0.125	0.022	−2.576	0.010	*
b	BMQ-Specific→BAASIS	−0.173	−0.310	0.026	−6.554	<0.001	***
c	SSRS→BAASIS	−0.049	−0.180	0.014	−3.503	<0.001	***
d	C-MLS→BAASIS	−0.179	−0.219	0.041	−4.392	<0.001	***
e	C-MLS→BMQ-specific	0.159	0.109	0.078	2.022	0.043	*
f	SSRS→BMQ-specific	0.177	0.363	0.026	6.761	<0.001	***
g	C-MLS→C-RESE	0.562	0.311	0.095	5.942	<0.001	***
h	SSRS→C-RESE	0.144	0.238	0.032	4.544	<0.001	***

Footnote: *p < 0.05, **p < 0.01, ***p < 0.001, *β* = Standardized coefficient, *B* = Unstandardized Coefficients; SE, standard error; CR, critical ratio, C-MLS, Chinese Version of the Medication Literacy Scale; SSRS, social support rate scale, BMQ-Specific = Beliefs about Medicines Questionnaire-specific; C-RESE, Chinese Version of the Regulation Self-efficacy Scale; BAASIS, Basel Assessment of Adherence with Immunosuppressive Medication Scale.

Mediation effects were further tested using the bootstrap method with 5000 resamples. A significant mediation effect was confirmed when the 95% confidence intervals for the total, direct, and indirect effects did not include zero (Byrne, 2016). The results in Table 7 indicate the following:

**TABLE 7:** Mediation effect tests for structural equation modeling (n = 351).

No.	Paths	Effect types	*β*	95%*CI*		*P*	Efficiency ratio
				Lower	Upper		
1	C-MLS→BMQ-Specific→BAASIS	Indirect effect	−0.034	−0.070	−0.002	0.039	0.116
	C-MLS→ C-RESE→BAASIS	Indirect effect	−0.039	−0.079	−0.010	0.007	0.134
	C-MLS→BAASIS	Direct effect	−0.219	−0.320	−0.114	0.001	0.750
	C-MLS→BMQ-specific/C-RESE→BAASIS	Total effect	−0.292	−0.387	−0.189	0.001	
2	SSRS→BMQ-Specific→BAASIS	Indirect effect	−0.113	−0.170	−0.065	<0.001	0.350
	SSRS→ C-RESE→BAASIS	Indirect effect	−0.030	−0.063	−0.008	0.007	0.093
	SSRS→BAASIS	Direct effect	−0.180	−0.275	−0.083	0.001	0.557
	SSRS→BMQ-specific/C-RESE→BAASIS	Total effect	−0.323	−0.404	−0.242	<0.001	

Footnote: *β* = Standardized coefficient, C-MLS, chinese version of the medication literacy scale; SSRS, social support rate scale, BMQ-Specific = Beliefs about Medicines Questionnaire-specific, C-RESE, Chinese Version of the Regulation Self-efficacy Scale; BAASIS, Basel Assessment of Adherence with Immunosuppressive Medication Scale.

In the effect of medication literacy on medication adherence, both medication beliefs and RESE showed significant mediation. Medication beliefs accounted for 11.6% of the effect (indirect effect β = −0.034, 95% CI: −0.070 to −0.002, *p* = 0.039), while RESE accounted for 13.4% (indirect effect β = −0.039, 95% CI: −0.079 to −0.010, *p* = 0.007). The mediating role of medication beliefs was stronger than that of RESE.

In the effect of social support on medication adherence, medication beliefs also demonstrated significant mediation, accounting for 35.0% of the effect (indirect effect β = −0.113, 95% CI: −0.170 to −0.065, *p* < 0.001). RESE contributed 9.3% (indirect effect β = −0.030, 95% CI: −0.063 to −0.008, *p* = 0.007). The magnitude of mediation was comparable between the two factors.

## 4 Discussion

This study revealed that medication adherence among KTRs remains suboptimal, with 37.6% of participants exhibiting non-adherent behavior. The most prevalent type was dosing irregularities (33.3%). More importantly, grounded in the COM-B model, this study demonstrated that medication literacy (capability) and social support (opportunity) not only directly served as negative predictors of non-adherence but also indirectly improved medication adherence through the dual mediating pathways of medication beliefs and RESE (motivation). The identification of this multiple mediation mechanism provides a critical theoretical basis for developing multidimensional and tailored intervention strategies.

Numerous studies have reported that the prevalence of IM non-adherence among KTRs ranges from 20% to 70%, which may be attributed to variations in measurement tools and assessment criteria (Paterson et al., 2018; Gokoel et al., 2020; Zhou et al., 2025; Mathes et al., 2017). In the present study, 37.6% of participants (132 cases) reported IM non-adherence, a rate lower than previously documented. All participants were recruited from a single center with a long-term focus on post-transplant adherence research and consistent provision of health education. This ongoing educational support may contribute to improved medication adherence in our cohort.

Among IM non-adherence behaviors, the most common is taking medication more than 2 hours outside the prescribed time (33.3%), followed by missing doses entirely (20.8%). Medication non-adherence can be categorized as either unintentional or intentional. Studies indicate that unintentional non-adherence, often due to forgetting, is more common among KTRs (Griva et al., 2018). In contrast, lower self-efficacy in heart transplant recipients is associated with higher rates of intentional non-adherence, and concerns about adverse drug effects may also lead patients to intentionally skip doses (Marston et al., 2023). To improve medication adherence in KTRs, healthcare providers should adopt standardized measures for adherence screening, actively identify underlying reasons for non-adherence, and implement targeted health education and behavioral interventions to enhance IM compliance.

Of note in this study, among the 117 patients with non-adherent medication behavior, 64 (54.7%) had received a living donor kidney, 76 (64.9%) had undergone pre-transplant dialysis for less than 1 year, 116 (99.2%) were taking more than three immunosuppressive medications, and 111 (94.9%) had a post-transplant time exceeding 1 year. These findings align with the regression analysis performed in this study, potentially revealing underlying cognitive and belief-related drivers of non-adherence, specifically that:

1. Donor Type and Risk Perception: This study found that recipients of deceased donor kidneys demonstrated better medication adherence than those receiving living donor transplants. Within China’s context of organ scarcity, deceased donor recipients may develop a stronger sense of appreciation and responsibility (motivation-M) toward their “hard-won” organs (Chen L. et al., 2023; Wang et al., 2020; Denhaerynck et al., 2014). In contrast, the shorter waiting times and generally favorable outcomes associated with living donor transplantation may foster unrealistic expectations of success among some recipients, leading them to underestimate long-term risks and weaken their belief in the necessity of medication, thereby reducing adherence to immunosuppressive therapy (Basiri et al., 2020). Thus, structured continuity-of-care education—including risk quantification and regular cognitive assessment—should be provided to living donor recipients to strengthen their medication beliefs. Additionally, this study observed that living donor recipients tended to be younger and less educated, a finding consistent with previous research by Denhaerynck et al. (2014). Therefore, healthcare providers should prioritize continuous education for this group, aiming to enhance risk awareness, medication beliefs, cognitive levels, and social support to effectively improve adherence.
2. Dialysis Experience and Avoidance Motivation: Patients with a dialysis history of ≥1 year demonstrate better medication adherence. Hemodialysis and peritoneal dialysis are primary treatments for end-stage renal disease; however, patients often experience complications such as fatigue, anemia, infection, and electrolyte disturbances. Long-term dialysis can also impair the cardiovascular system, leading to conditions like hypertension and coronary artery disease, with a mortality risk 10 to 20 times higher than that of the general population (McIntyre, 2024). A systematic review by Tang et al. (2021) indicated that recipients with longer dialysis duration adhere more strictly to their medication regimen—even in the presence of adverse effects—due to fear of returning to dialysis. These findings suggest that healthcare providers should implement stratified interventions: for those with <1 year or no dialysis history, focus on strengthening medication beliefs and behavioral support; for those with >1 year of dialysis, emphasize prevention and management of cardiovascular complications. Such tailored approaches may synergistically improve outcomes and reinforce treatment confidence.
3. Medication complexity and implementation burden: KTRs taking three or fewer medications generally demonstrate better adherence. Complex regimens increase the demands on patients’ functional ability (capability), raising the likelihood of missed or incorrect doses and potentially triggering psychological resistance (Tesfaye et al., 2024; Seng et al., 2020). Peng et al. (2022) found that among patients already taking more than ten medications, each additional drug increased the risk of non-adherence by 59.1%. Extensive medication lists require significant patient effort to understand and follow, often leading to dosing errors and negative emotional responses. However, Couzi et al. (2013) reported that patients on more medications sometimes showed better adherence, possibly due to a stronger belief in the necessity of treatment. Therefore, regardless of the number of medications, healthcare providers should prioritize patient education and psychological support. For those on fewer drugs, it is important to prevent underestimation of disease severity. For patients on complex regimens, clinical teams, including physicians and pharmacists, should aim to simplify the regimen (Opportunity) when possible by eliminating nonessential medications while clearly explaining that the regimen can be gradually reduced as the condition stabilizes. This approach helps lower psychological burden and enhances motivation to adhere.
4. Time Post-Transplantation and Risk Desensitization: Medication adherence is highest within the first year after transplantation but declines significantly thereafter, a trend consistent with findings from multiple studies (Wang et al., 2020; Derejie et al., 2024). Long-term use of IM may expose patients to considerable symptomatic burden, which can adversely affect adherence (Kaplan et al., 2023). Research by Bertram et al. (2016) indicates that as time progresses, although patients’ quality of life improves, their knowledge regarding IM and perceived risk of rejection tend to diminish. This dulling of risk perception—particularly within the motivation domain—poses a major threat to long-term adherence. In qualitative interviews conducted by Ruppar and Russell (2009), long-term KTRs reported becoming overconfident in their medication routines, leading them to undervalue reminder tools and increasing the likelihood of taking doses early or late. Therefore, even for recipients with prolonged transplant survival, ongoing IM-related health education remains essential. Interventions should aim to reinforce awareness of rejection and graft loss risks, teach practical medication management skills, foster consistent dosing habits, and provide regular psychological support to alleviate fears related to graft failure. A comprehensive strategy integrating education, behavior, and emotional support shows promise in improving medication adherence in this population.

Notably, findings often vary depending on sample characteristics and observation time points. In contrast to some previous studies (Belaiche et al., 2017; Mikulski et al., 2022), demographic factors such as age and education level did not demonstrate significant influence in this study, highlighting the complexity of factors affecting IM adherence. However, compared to these relatively static demographic and disease-related variables, this study focuses on medication literacy, social support, medication beliefs, and RESE—factors that may be more modifiable and thus offer promising targets for tailored interventions. Based on the identified mediation pathways and effect sizes, we developed a clinical management framework (Table 8) that integrates assessment tools, COM-B diagnosis, and intervention strategies, providing a structured approach for systematically identifying and addressing medication non-adherence.

**TABLE 8:** A clinical framework for assessing and intervening in medication non-adherence based on the COM-B model and study findings.

COM-B component	Core issue	Recommended clinical assessment tool	Intervention goal	Targeted intervention strategies
Motivation (medication beliefs/Emotional self-efficacy)	Belief issue: Insufficient understanding of the necessity of medication or excessive concern about side effects	Beliefs about medicines questionnaire-specific (BMQ-specific)	Improve medication knowledge and treatment understanding	• Motivational interviewing to explore and address medication concerns and shift negative beliefs • Shared decision-making to enhance treatment engagement • Personalized belief assessment and targeted plan development • Focus on reinforcing risk awareness (especially for living donor kidney recipients and long-term post-transplant patients)
	Emotional issue: Lack of confidence in managing negative emotions related to medication (e.g., depression, anxiety)	Regulatory emotional self-efficacy scale (RESE)	Enhance the ability to cope with emotional fluctuations	• Cognitive behavioral therapy to identify and restructure irrational thoughts • Emotional regulation training (mindfulness, relaxation techniques, stress management, etc.) • Provide anticipatory guidance on emotional fluctuations caused by medication side effects, and prepare coping strategies for emotional ups and downs • Family member support guidance (encourage effective communication and emotional expression)
Capacity (medication literacy)	Knowledge/Skill issue: Inability to correctly understand, calculate, or manage complex medication regimens	Medication literacy scale (MLS)	Improve medication knowledge and treatment understanding	• Regular medication education courses • Personalized medication education (for patients, family members, low education levels, using simple language) • Medication management skill training (e.g., using pill boxes, creating checklists) • Assess and ensure patients’ core abilities in calculating doses and understanding instructions
Opportunity (social Support)	Environmental/Social issue: Lack of effective reminders and support from family, friends, or the healthcare system	Social support rating scale (SSRS)	Strengthen family, peer, and healthcare support	• Family-involved care, training family members to become medication assistants • Establish peer support systems (e.g., patient support groups) • Guide patients to proactively seek help and increase their utilization of support • Simplify medication regimens while ensuring therapeutic efficacy (especially for those with multiple medications)

It should be noted that the medication adherence score in this study was reverse-scored, meaning that lower scores indicate better actual adherence. Therefore, when interpreting regression or correlation coefficients related to adherence, a negative value signifies a positive influence, whereas a positive value indicates a negative influence.

The findings demonstrate that medication literacy can directly influence medication adherence and also exert indirect effects through recipients’ RESE and medication beliefs. Among these, the direct effect of medication literacy on adherence was the largest (75.0%). The total mediating effect of medication beliefs and RESE accounted for 25.0%. In the parallel mediation pathways, the mediating effect of medication beliefs accounted for 11.6%, which was similar to that of self-efficacy in emotional regulation (13.4%). The specific pathways are as follows:

### 4.1 The direct effect of medication literacy on adherence

Medication literacy in KTRs demonstrated a significant direct negative effect on IM adherence (*β* = −0.219, 95% CI: −0.320 to −0.114, *p* = 0.001). This direct effect accounted for 75.0% of the total effect, indicating that it operates primarily through a direct pathway. Higher medication literacy was associated with better adherence, a finding consistent with studies in other patient populations. Patients with insufficient medication literacy are more prone to non-adherence, often due to an inability to correctly comprehend medication information or unmet treatment expectations. Conversely, those with high literacy proactively seek knowledge; their in-depth understanding of the treatment regimen improves acceptance and motivates regular therapeutic drug monitoring, thereby effectively mitigating risks of rejection and infection (Ng et al., 2022; Zheng et al., 2019; Tuo et al., 2023). Furthermore, Luo et al. (2023) reported that in adolescent populations, inadequate medication literacy significantly prolongs the disease transition period and increases the risk of medication errors. Therefore, systematically enhancing patients’ medication literacy should be a foundational interventional strategy for improving medication adherence and long-term outcomes.

### 4.2 The indirect effect of medication literacy on medication adherence: medication literacy → medication beliefs → medication adherence

Medication beliefs partially mediated the relationship between medication literacy and adherence among KTRs (*β* = −0.034, 95% CI: −0.070 to −0.002, *p* = 0.039), accounting for 11.6% of the total effect. Medication beliefs reflect an individual’s evaluation of the necessity of treatment versus concerns about adverse effects, representing a general attitude toward medication use. This pathway has been validated across multiple studies (Eser and Çelik, 2022; Neiva Pantuzza et al., 2022; Zhang et al., 2024): patients’ perceptions directly influence medication-taking behaviors. Insufficient appreciation of treatment benefits or excessive worry about side effects can lead to intentional non-adherence, such as self-directed dose reduction or discontinuation. Inadequate medication knowledge may stem not only from previous negative experiences but also from misunderstandings regarding the long-term value of treatment, which can amplify concerns about potential harms. Therefore, in clinical management of KTRs, healthcare providers should reinforce medication guidance and improve patients’ ability to understand and manage drug-related information through systematic education. Interventions should also focus on modifying medication beliefs by clearly emphasizing the necessity and importance of consistent immunosuppressive therapy while proactively addressing concerns about side effects and strengthening perceptions of treatment benefits to ultimately promote behavioral adherence.

### 4.3 Indirect effect of medication literacy on medication adherence: medication literacy → RESE → medication adherence

RESE partially mediated the relationship between medication literacy and medication adherence (*β* = −0.039, 95% CI: −0.079 to −0.010, *p* = 0.007), accounting for 13.4% of the total effect, which was comparable to the mediating effect of medication beliefs (11.6%). This pathway can be explained by the fact that patients with higher medication literacy are generally better able to rationally evaluate and actively manage emotional distress arising from lifelong medication use, financial burden, complications, and complex treatment regimens (Harrington and Morgan, 2016; Chu et al., 2020). Supporting this, Wang et al. (2024) noted that improved medication literacy helps KTRs proactively seek support and enhance psychological resilience, thereby regulating negative emotions more effectively and reducing non-adherence. These findings suggest that clinical interventions should go beyond standard medication education to include proactive guidance on anticipated emotional side effects and targeted psychological support. By systematically strengthening patients’ RESE, medication literacy can be effectively translated into improved medication adherence.

The findings demonstrate that social support directly influences medication adherence and also exerts indirect effects through recipients’ self-regulation efficacy and medication beliefs. The direct effect of social support accounted for the largest proportion (55.7%) of the total effect on adherence. In contrast, the total mediating effect of medication beliefs and self-regulation efficacy represented 44.3%. Among the parallel mediation pathways, medication beliefs contributed the most (35.0%), while self-regulation efficacy accounted for only 9.3%. The specific pathways are detailed below.

### 4.4 The direct effect of social support on medication adherence

Social support had a significant direct negative effect on IM adherence among KTRs (*β* = −0.180, 95% CI: −0.275 to −0.083, *p* = 0.001). The direct effect accounted for 55.7% of the total effect, indicating its substantial role in the overall relationship. This finding aligns with the results reported by Ladin et al. (2018), who observed that higher levels of social support were associated with better IM adherence in KTRs.

The beneficial effect of social support on adherence may vary by its form. As highlighted in a systematic review by Ladin et al. (2018), perceived support is generally associated with higher medication adherence, whereas enacted support tends to have a more limited impact. Consistent with this, our study found that the subjective support dimension scored the highest (22.99 ± 5.07) within participants’ social support structure. Subjective social support, as an internal positive psychological resource, can influence external behavior and long-term personal development and may be more predictive and functionally relevant than enacted support (Hu et al., 2023). Specifically, medication reminders and emotional companionship from family and friends can make patients feel cared for and valued, thereby motivating them to maintain medication behaviors expected by others (Yang et al., 2022; Chironda and Bhengu, 2019; Hong et al., 2017).

Beyond support from family and friends, peer support from other transplant recipients with similar experiences represents another valuable form of social support (Jamieson et al., 2016). Therefore, healthcare providers may enhance KTRs’ social support levels by organizing online and in-person communication activities to facilitate peer interactions and foster sustainable peer support networks.

### 4.5 The indirect effect of social support on medication adherence: social support → medication beliefs → medication adherence

Medication beliefs partially mediated the relationship between social support and medication adherence (*β* = −0.113, 95% CI: −0.170 to −0.065, *p* < 0.001), accounting for 35.0% of the total effect. This indicates a substantial mediating role of medication beliefs in the model. The findings are consistent with research by Gong et al. (2024), which suggested that high levels of social support can enhance patients’ perceptions of medication necessity and strengthen their confidence in managing illness, thereby promoting healthier behaviors. Specifically, through explaining the importance of medication, offering emotional support, and sharing health-related information, families and healthcare teams can reinforce patients’ treatment acceptance and reduce medication-related concerns.

However, as Been-Dhamen et al. (2018) observed, although many patients desire more professional support, the reality is that a “one-size-fits-all” approach to patient education often fails to address individual needs. In situations involving complex medication-related risks, an imbalance in patient-provider power dynamics may discourage patients from voicing concerns, thereby directly hindering adherence (Montori et al., 2023; Hole et al., 2023).

These findings emphasize the need of using tailored belief assessments in clinical follow-ups to detect individuals with serious medication issues. To help patients communicate medication concerns, joint decision-making and motivational interviewing are recommended. Based on these judgments, provide tailored information guidance. Structured health education for patients’ relatives is also necessary to improve post-transplant drug comprehension. These efforts can help families provide more precise and effective medication support, boosting drug adherence (Xiong et al., 2023).

### 4.6 The indirect effect of social support on medication adherence: social support → RESE → medication adherence

The indirect effect of social support on medication adherence through RESE was significant (*β* = −0.030, 95% CI: −0.063 to −0.008, *p* = 0.007), indicating partial mediation with an effect size of 9.3%. This aligns with previous research demonstrating that social support can indirectly influence medication adherence in transplant recipients via RESE (Guo et al., 2015; Gong et al., 2024).

However, as Lin et al. (2015) observed, most KTRs recover at home post-transplantation and rely primarily on family support. Financial strain from treatment costs may lead family members to express stress unintentionally, evoking guilt or self-blame in patients. Such emotions can result in missed doses as patients attempt to reduce household expenses (Wurm et al., 2022; Al-Talib et al., 2024). While family involvement provides essential practical and emotional support, communication focused on economic burden may undermine its benefits.

Thus, while urging patients to seek social support, healthcare providers can also help family members build RESE—the ability to recognize, accept, and actively control negative emotions. Before discharge, families should be advised to avoid overemphasizing treatment expenditures in front of patients and instead focus on emotional wellbeing and open communication and expression. Patients should also learn emotion-regulation skills like mindfulness and cognitive restructuring to self-adjust under stress and maintain medication adherence with external help. (Bulbuloglu and Gunes, 2024; Saritas et al., 2025).

This study has several limitations. First, as a single-center cross-sectional survey, all participants were recruited from a single transplant center in Changsha, Hunan Province. Second, we only included patients with a functional graft to focus on behavioral and psychological factors in those with preserved renal function. However, this criterion may have resulted in selection bias by excluding individuals who experienced graft loss as a consequence of extreme non-adherence. Third, self-reported measures may overestimate actual medication adherence. Both intentional and unintentional non-adherence were assessed solely via self-report, which is inherently susceptible to bias due to social desirability—such as concealing true medication behaviors to avoid criticism from healthcare providers (Adams et al., 1999; Tong et al., 2011; Stirratt et al., 2015). Additionally, recall bias represents another limitation of this approach (Hardstaff et al., 2003).

## 5 Conclusion

Medication adherence to immunosuppressants among KTRs remains suboptimal, with non-adherence to dosing schedules being the most prevalent issue. Donor type, dialysis history, medication complexity, and time since transplantation significantly influence adherence. Based on the COM-B framework, this study revealed that medication literacy (capability) and social support (opportunity) not only directly affect adherence but also indirectly improve it by strengthening medication beliefs and RESE. However, given the study’s limitations, future research should adopt multicenter, large-sample longitudinal designs. Such approaches would enhance the robustness of findings and provide sufficient statistical power for in-depth subgroup analyses (e.g., living vs. deceased donor recipients, patients at different post-transplant stages), thereby validating the model’s generalizability and informing targeted interventions. These studies should combine objective measures (e.g., eGFR, rejection episodes, tacrolimus/cyclosporine trough levels, electronic monitoring) with subjective reports to comprehensively evaluate adherence.

## Data Availability

The original contributions presented in the study are included in the article/Supplementary Material, further inquiries can be directed to the corresponding author.

## Abbreviations

KT, Kidney transplantation; KTRs, Kidney transplant recipients; IM, Immunosuppressive Medication; RESE, Regulatory Emotional Self-Efficacy; COM-B, Capacity, opportunity, motivation-behavior; WHO, the World Health Organization; KMO, Kaiser-Meyer-Olkin Measure of Sampling Adequacy; S-CVI, Scale-Level Content Validity Index; C-MLS, Chinese Version of the Medication Literacy Scale; SSRS, Social Support Rating Scale; BMQ-Specific, Beliefs about Medicines Questionnaire-Specific; C- RESE, Chinese Version of the Regulatory Emotional Self-Efficacy Scale; POS, Positive emotions; DES, Despondency/distress; ANG, Anger/irritation; BAASIS:Basel Assessment of Adherence with Immunosuppressive Medications Scale; VIF: Variance Inflation Factor; SEM:Structural equation modeling; 95% CI: 95% confidence intervals; M: Mean,; SD: standard; β: Standardized coefficient; B: Unstandardized Coefficients,; SE: Standard Error; CR: Critical Ratio.
