# Supplementary material for: Investigating the pathways of medication adherence in renal transplant recipients based on the COM-B model: the parallel mediating effects of beliefs and emotion regulation efficacy
Source: Front Pharmacol. 2025 Dec 1;16:1702618. doi: 10.3389/fphar.2025.1702618 (PMC12702757; doi:10.3389/fphar.2025.1702618)
Supplement: Supplementary file 1 [file DataSheet1.pdf]

Supplementary Table S1: Normality Test Results of Included Variables

Variable	Skewness	Kurtosis
Medication Literacy	-0.474	-1.364
Medication Beliefs	-0.236	-0.539
Emotional Self-Regulation Efficacy	-0.220	-0.545
Social Support	-0.101	-0.682
Medication Adherence	2.305	5.582

Supplementary Table S2: Multicollinearity Test

Variable		Model 1		Model 2		Model 3		Model 4		Model 5	
		Tolerance	VIF	Tolerance	VIF	Tolerance	VIF	Tolerance	VIF	Tolerance	VIF
Gender		0.983	1.017	0.980	1.020	0.973	1.027	0.971	1.030	0.971	1.030
Kidney transplant source		0.670	1.493	0.648	1.544	0.636	1.573	0.634	1.577	0.612	1.634
Number of transplants		0.970	1.031	0.965	1.037	0.964	1.037	0.964	1.038	0.964	1.038
Pre-Transplant Dialysis Duration		0.870	1.150	0.870	1.150	0.859	1.164	0.843	1.186	0.812	1.232
Medication Types		0.979	1.021	0.979	1.021	0.979	1.021	0.979	1.022	0.976	1.025
Postoperative Complications		0.648	1.544	0.648	1.544	0.638	1.568	0.633	1.581	0.620	1.613
Post-Transplant Duration	< 1 year	0.547	1.829	0.536	1.865	0.534	1.871	0.528	1.893	0.527	1.899
	1-5 years	0.536	1.867	0.529	1.892	0.521	1.919	0.516	1.938	0.504	1.983
	5-10 years	0.575	1.740	0.574	1.741	0.566	1.765	0.565	1.770	0.562	1.779
Medication Beliefs				0.898	1.114	0.868	1.152	0.786	1.272	0.784	1.275
Emotional Self-Regulation Efficacy						0.830	1.205	0.779	1.283	0.748	1.338
Social Support								0.703	1.422	0.665	1.504
Medication Literacy										0.597	1.675

Footnote：VIF =Variance Inflation Factor

Supplementary Table S3：Classification of Postoperative Complications

Category	Specific Examples (Including but not limited to)
**Infection**	Tuberculosis, Pneumocystis jirovecii Pneumonia, Cytomegalovirus infection
**Cardiovascular Diseases**	Hypertension, Hyperlipidaemia, Coronary heart disease, Deep vein thrombosis
**Endocrine and Metabolic Disorders**	Post-transplantation diabetes mellitus, Hypercalcaemia, Hyperuricaemia, Hypophosphataemia, Metabolic bone disease
**Drug-induced Liver Injury**	Abnormal liver function caused by immunosuppressants or other related medications
**Neuropsychiatric Impairment**	Mood disorders, Anxiety, Depression, Tremor, Ataxia
**Malignancy**	Various types of newly developed tumours post-transplantation
**Ocular Complications**	Cataracts, Glaucoma
**Others**	Cushing's syndrome facies (moon face), Osteoporosis, Gingival hyperplasia

Supplementary Table S4. Differences in Medication Adherence by Sociodemographic Characteristics of the Study Participants（n=351，x̄±s）

Variabes	N (%)	*M±SD*	*t/F/H*	*P*	*Tamhane T2*
Gender					
Male	200 (57.0)	6.24±2.00	2.027(2)	**0.043**	
Female	151 (43.0)	5.83±1.69			
Age					
18-44	147 (41.9)	6.06±1.67	0.166(1)	0.847	
45-59	143 (40.7)	6.01±1.99			
≥ 60	61 (17.4)	6.18±2.09			
Education level					
Junior school or below	112 (31.9)	6.36±2.11	2.172(1)	0.091	
High school	103 (29.3)	6.05±1.80			
Junior college	66 (18.8)	6.05±1.86			
Bachelor degree or above	70(19.9)	5.63±1.59			
Marital status					
Married	301 (85.8)	6.09±1.90	0.577(2)	0.564	
Unmarried/Divorced/Widowed	50 (14.2)	5.92±1.81			
Employment status					
Employed	109 (31.1)	6.25±2.08	2.708(1)	0.468	
Retired	67 (19.1)	5.99±1.98			
Unemployed	175 (49.9)	5.98±1.72			
Kidney transplant source					
Living relative donor	154 (43.9)	6.76±2.35	6.005(2)	**＜0.001**	
Hospital donation	197 (56.1)	5.52±1.16			
Number of transplants					
Once	332 (94.6)	6.09±1.93	2.748(2)	**0.010**	
> Once	19 (5.4)	5.53±0.77			
Post-transplant duration (years)					
< 1	83 (23.6)①	5.20±0.75	19.760(3)	**＜0.001**	①＜②***, ①＜③***,
1-5	114 (32.5)②	5.77±1.06			①＜④***, ②＜③*,
5-10	85 (24.2)③	6.40±1.95			②＜④**

> 10	69 (19.7)④	7.10±2.96		
Pre-transplant dialysis duration (years)				
< 1	179 (51.0)	6.58±2.26	5.528(2)	**＜0.001**
≥ 1	172 (49.0)	5.52±1.18		
Number of medications				
≤ 3	19 (5.4)	5.26±1.15	-2.984(2)	**0.006**
> 3	332 (94.6)	6.11±1.91		
Daily medication frequency (doses)				
≤ 2	164 (46.7)	5.90±1.82	0.855(1)	0.464
3-4	148 (42.2)	6.19±1.92		
5-6	35 (10.0)	6.31±2.08		
≥ 7	4 (1.1)	6.00±1.41		
Postoperative complications				
Yes	250（71.2）	6.27±2.10	4.302(2)	**＜0.001**
No	101（28.8）	5.54±1.05		

Footnote: (1) = One-way ANOVA, (2) = Independent-samples *t* test, (3) = Kruskal-Wallis *H* test, *M* = Mean, *SD* = standard deviation, **p* < 0.05, ***p* < 0.01, ****p* < 0.001.
